# cΔlog k_w_^IAM^: can we afford estimation of small molecules’ blood-brain barrier passage based upon *in silico* phospholipophilicity?

**DOI:** 10.5599/admet.1034

**Published:** 2021-12-15

**Authors:** Lucia Grumetto, Giacomo Russo

**Affiliations:** 1 Pharm-Analysis & Bio-Pharm Laboratory, Department of Pharmacy, School of Medicine and Surgery, University of Naples Federico II, Via D. Montesano, 49, I-80131, Naples, Italy; 2 School of Applied Sciences, Sighthill Campus, Edinburgh Napier University, 9 Sighthill Ct, EH11 4BN Edinburgh, United Kingdom

**Keywords:** immobilized artificial membrane, biochromatography, blood brain barrier, brain targeting, phospholipophilicity

## Abstract

56 compounds, whose log BB values were known from the scientific literature, were considered and their phospholipophilicity values were calculated in silico. These values, along with either experimentally determined or calculated lipophilicity values, were used to extract cΔ/Δ’log *k*_w_^IAM^ parameters. cΔ/Δ’log *k*_w_^IAM^ values were found inversely related to data of blood-brain barrier passage, especially in the < -0.20 log BB range and on the IAM.PC.DD2 phase (r^2^ = 0.79). In multiple linear regression, satisfactory statistic models (r^2^ (n-1) = 0.76), based on cΔ/Δ’log *k*_w_^IAM.MG^ along with other in silico calculated descriptors, were achieved. This method brings the potential to be applied, along with other methodologies, to filter out solutes whose BBB permeation is foreseen to be substandard, thus allowing pharmaceutical companies/research institutes to focus on candidates that are more likely to concentrate in the brain.

## Introduction

Combinatorial chemistry involves the generation of a large array of structurally diverse compounds, *i.e.*, a chemical library, through systematic, repetitive and covalent linkage of various “building blocks” [1]. This technique can be exploited in parallel, delivering hundreds, if not thousands, of molecules of pharmaceutical interest in a handful of hours. While the organic synthesis throughput has expanded so noticeably in recent years, screening methodologies are still lagging behind, instead [2]. Indeed, most of the testing still requires animal models that have the undeniable advantage of mirroring more closely the complexity of human beings than cells. However, animal models are facing criticism from the public since they often require the sacrifice of vertebrates [3] and heavily impact the environment due to the huge number of carcasses to dispose of.

The assessment of the ability of a drug to cross the biological membranes in the early stages of its development plays a pivotal role in pharmaceutical industrial research. Notably, the development of drugs acting toward the central nervous system (CNS) has poorer success rates and requires longer times than non-CNS drugs [4]. This occurs due to the complexity of the blood-brain barrier (BBB).

In fact, in a healthy brain, the BBB plays a crucial role in protecting normal brain functions from potentially harmful compounds occurring in the bloodstream [5]. Strategies for brain drug delivery have developed in the last decades, and various techniques are available to study the BBB’s role in drug uptake. These include *in vivo*, *in vitro* [6] and *in situ* techniques [7].

Separation science offers valuable alternatives to animal testing that can provide effectiveness in the drug discovery/drug development pipeline as biomimetic liquid chromatography [8-11], performed employing stationary phases emulating biological components or using mobile phase ingredients simulating physiological environments. A consistent branch of this is represented by liquid chromatography (LC) conducted on stationary phases based on immobilized artificial membranes (IAM). IAM phases are based on membrane phospholipid analogs covalently bound to aminopropyl silica [7,12,13]. Some of these phases are available commercially as IAM.PC.MG and IAM.PC.DD2. Both these support phosphatidylcholine analogs (PC), but they differ from each other in the end-capping of the free aminopropyl moieties, which is performed with methyl glycolate (MG) or with C_3_ or C_10_ anhydrides (DD2).

In recent years, we parameterized the excess of the polar/electrostatic interactions occurring between drugs and biological membranes as Δlog *k*_w_^IAM^ [14-19]. Δlog *k*_w_^IAM^ is obtained by combining *n*-octanol/water lipophilicity with phospholipophilicity, i.e., the affinity of the compound for the IAM phases measured as a retention factor extrapolated at 100 % of aqueous phase (*k*_w_^IAM^) [20]. This represents the difference between the logarithm of the chromatographic retention factor (log *k*_w_^IAM^) measured for each analyte, i.e., the experimentally determined phospholipophilicity, and the value expected for a neutral isolipophilic molecule that is estimated by correlative equations. Δlog *k*_w_^IAM^ values were inversely related to the drug passage of complex biological barriers, such as the BBB and the intestinal wall [13,17,18]. The increasing need for high-throughput drug discovery methods has provided several *in silico* models of BBB permeation based on *in vivo* log BB values [21,22]. Log BB is generally measured on murine models and is still nowadays considered as a solid indication for BBB delivery [23]. Log BB is defined as (Eq. 1):


(1)

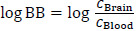



in which *C*_Brain_ is the concentration that the analyte realizes in the brain tissues, and *C*_Blood_ is the concentration that it achieves in the blood. The *in silico* models bring the advantages of being much faster to perform and applicable to molecules that are not yet synthesized and/or not easily detectable.

Back in 2017, we developed some statistical models to predict the phospholipophilicity of small molecules based on more than 200 individual measurements performed in our laboratories. This also materialized in an online service, namely log *k*_w_^IAM.MG/DD2^ calculator, offering the opportunity to predict the phospholipophilicity of all compounds included in PubChem collection as log *k*_w_^IAM^ on both MG and DD2 chromatographic columns [24].

In the present study, we aim at applying these statistical models to calculate the phospholipophilicity of a dataset of compounds whose log BB is known from the scientific literature and from there to estimate Δlog *k*_w_^IAM^, based either on experimentally determined or calculated lipophilicity values. Our goal is to evaluate whether these parameters calculated *in silico*, therefore called from here on cΔlog *k*_w_^IAM^, can offer effectiveness in screening libraries of compounds for their potential to reach the brain. If so, we will look at ways to implement these procedures in the drug discovery/development industrial programs.

## Experimental

### Data collection

Experimental lipophilicity values were collected from the scientific literature. Specifically, all the log *P* values were taken from PubChem but those of nevirapine and thioxolone, which were taken from the literature [25].

Calculated log *P* values were obtained by either ALOGPS [26] or by MarvinSketch [27]. For acidic compounds, whose Δ’log *k*_w_^IAM^ but not their Δlog *k*_w_^IAM^ were previously found related to log BB, log *D*^7.4^ values were again calculated by MarvinSketch software [27]. Log BB values were taken from the literature [28].

### cΔ/Δ’log k_w_^IAM^ values calculation

cΔ/Δ’log *k*_w_^IAM^ values were calculated from phospholipophilicity values estimated *in silico* according to a procedure we developed in 2017 [24]. In brief, the best relationships were found to be:


(2)

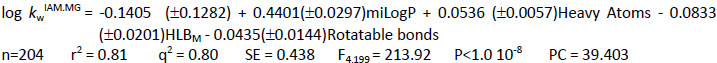



and


(3)

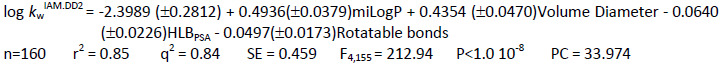



A detailed explanation of the main descriptors, along with relevant references, is reported in supporting information (Table S1). In these equations, *n* is the number of data considered to derive the regression equation, *r*^2^ is the square of the correlation coefficient, SE is the standard error of the estimate, F (the subscripts are the degrees of freedom and the number of variables) is the Fisher statistic of the regression, P is the observed significance level, i.e., the probability of obtaining a result equal to or “more extreme” than what was observed, and PC is the Amemiya predictive criterion of the regression.

The hydrophilic-lipophilic balance (HLB) can be taken into account by the methods by Griffin [29] (HLBG), Davies [30] (HLBD), and taking into account the steric effects (HLBPSA), not considered by the two approaches. HLBPSA is defined as follows:



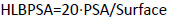



where PSA is the polar surface area and Surface is the total surface.

HLB (HLB_M_) is the mean resulting from the values by all three methods. miLogP is the octanol-water partition coefficient predicted by the online program for the calculation of molecular properties and bioactivity prediction [31].

The calculations were made completely automated and easily accessible to anyone via a user-friend tool to predict log *k_w_*^IAM.MG^ and log *k_w_*^IAM.DD2^, a Web service and a set of scripts for VEGA ZZ program [24]. This is available at https://www.ddl.unimi.it/vegaol/logkwiam.htm and offers a calculation of log *k*^IAM.MG/DD2^ of any molecule included in the PubChem collection as implemented in the script version.

Δlog *k*_w_^IAM^ values were calculated as the difference between the log *k*_w_^IAM^ computed from equations (2) and (3) and the log *k*_w_^IAM^ expected for neutral isolipophilic molecules. Indeed, as reported in our previous studies [14,15], IAM retention data on both IAM phases relate unambiguously with log *P* values of structurally non-related neutral compounds, in the log *P* range 1.0–4.8. These relationships are expressed by the following equations:


(4)

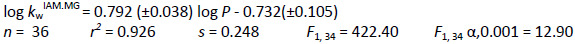




(5)

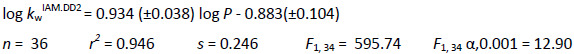



For acidic compounds, analogously to what was reported in our previous study [15], log *D*^7.4^ rather than log *P*^N^ was used for the computation of delta values in equations (4) and (5). Their values were therefore named w’log *k*_w_^IAM^ values to avoid any ambiguity.

### Molecular modeling

An ample array (> 1,600) of physico-chemical descriptors, subdivided into 20 logical blocks (atom type, functional group, fragment counts, topological and geometrical descriptors), were calculated by the web service E-DRAGON 1.0 [32]. In brief, the molecules were input as SMILES code in a text document and converted by the integrated applet CORINA in 3D before all the indices were computed. The Quantitative structure-property relationship (QSPR) models were obtained by the automatic stepwise approach implemented in the “automatic linear regression” script of VEGA ZZ software [33], calculating regression models, including from one to five independent variables. The predictive strength of the best equation was evaluated by leave-one-out (LOO) cross-validation. The regression coefficients were calculated to evaluate the set in terms of the standard deviation of errors (SE), regression coefficients (*r*^2^ is the square of the correlation coefficient, q^2^ is the square of the correlation coefficient after cross-validation), intercept, Fisher statistic for the regression (F), probability (P) and Amemiya prediction criterion (PC). Descriptors with too low regression coefficient (r^2^ < 0.1) were excluded, and collinear descriptors were detected by evaluating the variance inflation factor (VIF) whose threshold value was set to 5.

### Data handling

Data were input in a spreadsheet and data points were plotted from Microsoft Excel, part of the Microsoft Office 365 suite of programs.

## Results and Discussion

### cΔ/Δ’log k_w_^IAM^ : simple linear regression

In our previous studies [14-19, 34], Δ/Δ’log *k*_w_^IAM^ values were found inversely related to the passage of complex biological barriers, such as the BBB and the intestinal wall. The calculation of Δ/Δ’log *k*_w_^IAM^ parameters are based on two physico-chemical properties, i.e., *n*-octanol/water lipophilicity either of the neutral species (giving Δlog *k*_w_^IAM^) or of the mixture of the species at the physiological pH, i.e., 7.4 (giving Δ’log *k*_w_^IAM^) and the affinity of the compound for IAM phases. Indeed, in our previous studies [14-16], we verified that for acidic compounds, significant relationships *vs.* log BB data could only be obtained when delta parameters were calculated by using the lipophilicity of the mixture of the species present in solution at the experimental pH, i.e., log *D*^7.4^, rather than that of the neutral species, i.e., log *P*^N^. For neutrals, bases and ampholytes, delta parameters were estimated by using log P^N^ values instead. For consistency, we extended the same approach to delta values surrogated *in silico* (cΔ/Δ’log *k*_w_^IAM^).

However, while there are plenty of tools available to surrogate log *P* values [35], to the best of our knowledge, the *in silico* platform we developed is the only service that predicts phospholipophilicity. Table 1 lists names, chemical nature (A= acid, B=basic, BB= bibasic, N= neutral), calculated log *k*_w_^IAM.MG^ and log *k*_w_^IAM.DD2^, exp log *P*^N^, clog *P*^N^ and calculated log *D*^7.4^ (for acids only) values for the dataset considered. cΔ/Δ’log *k*_w_^IAM^ values are reported in Table 2 along with the experimental log BB values.

**Figure 1.**
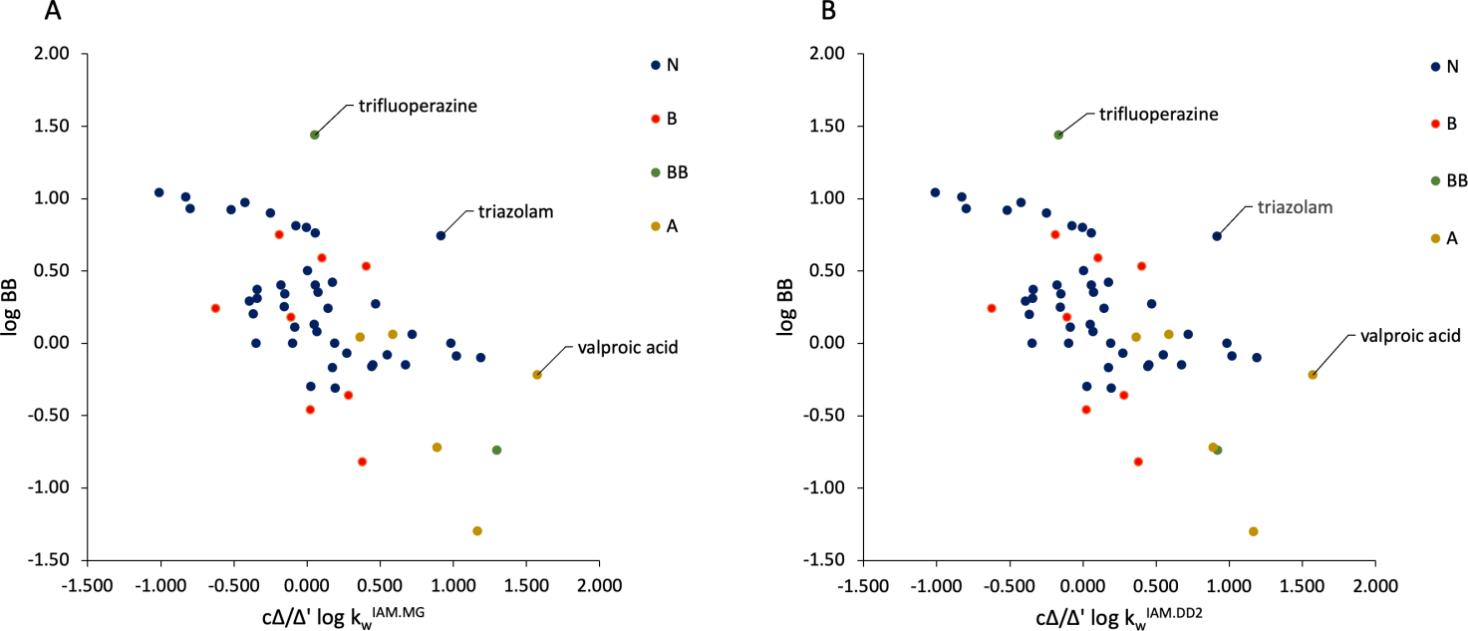
Relationships between log BB values and cΔ/Δ’log *k*_w_^IAM.MG^ (**A**) and cΔ/Δ’log *k*_w_^IAM.DD2^ values (**B**).

Figure 1 illustrates the relationships between log BB and the cΔ/Δ’log *k*_w_^IAM^ values on the MG (A) and DD2 (B) stationary phases and a clear descending trend is visible in both plots. These values, calculated by considering exp log *D*^7.4^ values for acids and log *P*^N^ values for all the other molecules, are reported in Table 2 along with the experimental log BB values. The dataset was divided according to the molecules’ ionization in neutrals (N), bases supporting one (B) or two (BB) basic groups and acidic (A) compounds. This was set to evaluate whether any specific trend was visible in each subgroup. Three of the assayed molecules markedly deviate from the pattern identified by the main distribution of points are triazolam, trifluoperazine and valproic acid. The chromatographic behaviorur of small molecules on IAM phases has been characterized by many research groups for more than three decades [38,39]. Trifluoperazine is a highly lipophilic base, and it is well ascertained [14] that these interact with phospholipids weaker than isolipophilic neutral compounds, especially on the IAM.PC.DD2 phase. As to triazolam and valproic acid, the reasons for these deviating from the main distribution of points do not seem that straightforward to spot. Triazolam is a benzodiazepine derivative featuring a structure of three condensed rings covalently bound to one chlorobenzene moiety sharing the same plane. It has been again already characterized [39] that those planar structures tend to interact with IAM phases stronger than the extent expected based on their lipophilicity, but it is hard to assess whether this played a role in this instance. For its being an acid, the calculation of cΔ’log *k*_w_^IAM^ of valproic acid was based on log *D*^7.4^ rather than log *P*^N^. However, since we could not retrieve the experimental value from literature sources, we had to rely on the calculated value, whose closeness to the actual value cannot be reasonably taken for granted. Interestingly, a descending trend is visible for neutral compounds between the cΔ/Δ’log *k*_w_^IAM^ and log BB values ranging from +1 to 0 but the distribution flattens for log BB < 0.

**Figure 2.**
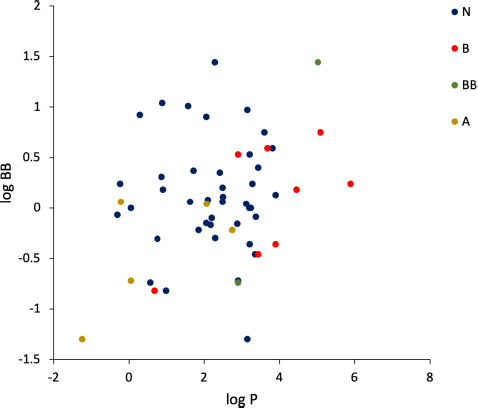
Relationships between log BB values and log *P* values.

Figure 2 instead displays the relationship occurring between the data of permeation through the BBB and experimental *n*-octanol/water lipophilicity values. The experimental log *P* values for 1-chloro-2,2,2-trifluoroethane, 1-hydroxymidazolam, 3-methylhexane, 4-hydroxymidazolam, bretazenil, desmonomethylpromazine, fluroxene, isoflurane, *n*-heptane, *n*-hexane, nordiazepam, northioridazine, *n*-pentane, teflurane and trichloroethylene were not available and, therefore, calculated values were used instead. Clearly, log *P* represents an index of paramount importance in pharmaceutical discovery and development [40]. The assumption is that lead compounds should lie in a specific range of lipophilicity to be considered for further implementations. The expectation is that lipophilicity should be positively related with data of drugs’ passage through complex barriers, including the BBB [40]. However, the extremely scattered data points of Figure 2 evidence that no relationship between log *P* and log BB values can be observed. Likewise, no trend is visible between the two considered variables if all the compounds are considered. However, an ascending trend is visible for acidic compounds, albeit their number is limited.

**Figure 3.**
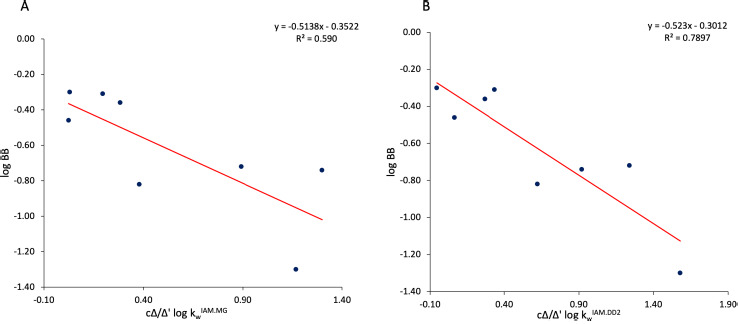
Relationships beween log BB values (<0.20) and cΔ/Δ’log *k*_w_^IAM.MG^ (**A**) and cΔ/Δ’log *k*_w_^IAM.DD2^ values (**B**).

**Figure 4.**
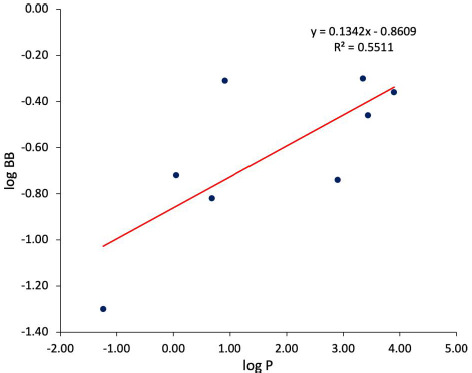
Relationships beween log BB (<0.20) and log *P* values.

Conversely, the situation changes noticeably when considering only the lowest range of log BB (< -0.20). Indeed, as Figure 3 displays, the relationship between log BB and cΔ/Δ’log *k*_w_^IAM^ becomes inverse linear for this subset with a rather solid *r*^2^ value, i.e., > 0.59, with a superior accuracy afforded by delta values on the DD2 phase. This is analogous to what we achieved using delta values obtained from experimentally determined log *k*_w_^IAM^ values [14-19,34] instead of the calculated ones. We subsequently compared the performance in predicting log BB values of delta descriptors again *vs.* experimentally determined log P^N^ values (detailed in Figure 4). Although a direct linear relationship is observable between log BB (< -0.20) and log *P*^N^ values, its accuracy as assessed from *r*^2^ is inferior to that of the relationship developed from cΔ/Δ’log *k*_w_^IAM.DD2^ values. If cΔ/Δ’log *k*_w_^IAM^ are calculated from *in silico* rather than experimental log *P* data, the relationship between log BB (< -0.20) and cΔ/Δ’log *k*_w_^IAM^ values weaken, although not much, especially on the DD2 phase (*r*^2^= 0.68 by using clogP values calculated by MarvinSketch, data not shown).

Although the size of our dataset is relatively limited (*n* = 56), we can extract some interesting information from the results achieved. Specifically, the method for predicting cΔ/Δ’log *k*_w_^IAM^ cannot yet be used alone in the discovery phase. However, this can be run as complementary along with other assays for profiling the ADME potential of drug candidates as it can provide additional information that is not afforded by other early assessments, e.g., lipophilicity. Moreover, the method hereby reported seems to be more selective in the identification of the candidates with the slimmest chances to gain access to the brain. This is advantageous, especially if the potency of the candidates that are screened is high enough to be effective, even if the amounts that are successfully delivered to the brain are low.

These considerations would support the implementation of this method as a filter in the discovery phase to filter out the compounds intended to act toward the brain, with substandard potential to partition in the CNS. The method seems to work better if the estimation of cΔ/Δ’log *k*_w_^IAM^ relies on experimental lipophilicity data rather than calculated ones. This is not an obstacle since many high throughput platforms for log *P* assessments are now available on the market [41] or being described [42] and for sure log *k*_w_^IAM^ measurements are more demanding since they require samples to be run over (at least) three organic modifier concentrations.

A further consideration concerns the models used to calculate phospholipophilicity. These are already rather good but could be improved by analysing more and more chemically diverse solutes to broaden their applicability space.

### cΔ/Δ’log k_w_^IAM^ : multiple linear regression

The passage of therapeutics through the BBB is unanimously recognized as an extremely complex phenomenon, which results from an interplay of various passage patterns, including transcellular passive, transcellular active and paracellular passage pathways [43]. Therefore, it is rather unlikely that a sole descriptor can encode all the interactions taking place in BBB uptake. For this reason, we calculated an ample array (> 1,600) of physico-chemical descriptors by the software E-DRAGON 1.0 and studied them in (i) simple linear regression and (ii) multiple linear regression *vs.* log BB values. Task (i) was done to establish how cΔ/Δ’log *k*_w_^IAM^ indexes compared to other physico-chemical descriptors in terms of predictive strength, while task (ii) was accomplished to study whether using multiple variables to model the BBB passage of the dataset could yield some useful statistic models.

The results of the simple linear regression analysis are listed in Table 3, along with the relevant statistics. An analysis of the data suggests that all regression coefficients are significant at the 99 % level. Among all the E-DRAGON descriptors, cΔ/Δ’log *k*_w_^IAM.DD2^ and cΔ/Δ’log *k*_w_^IAM.MG^ ranked fourth and fifth, respectively and their *r*^2^ values were exceeded only by parameters referring to polarity (TPSA(NO)), molecular lipophilicity (AlogPS), and the number of oxygen atoms (nO). A detailed explanation of these and relevant references is reported in supporting information (Table S1). The aspect that both the topological surface area and the number of oxygen atoms relate to a fair extent with the BBB passage of the molecules in the dataset may suggest that H-bonding may act by preventing the uptake of these chemicals through the BBB. This agrees well with the observations made by Diamond and co-workers [44] and other research groups [45].

Subsequently, the incorporation of cΔ/Δ’log *k*_w_^IAM^ descriptors was attempted in multiple linear regression reported below:


(6)

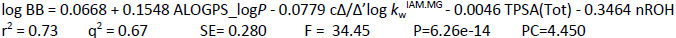



and after LOO:


(7)

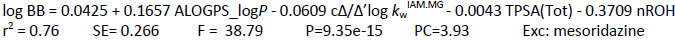



**Figure 5.**
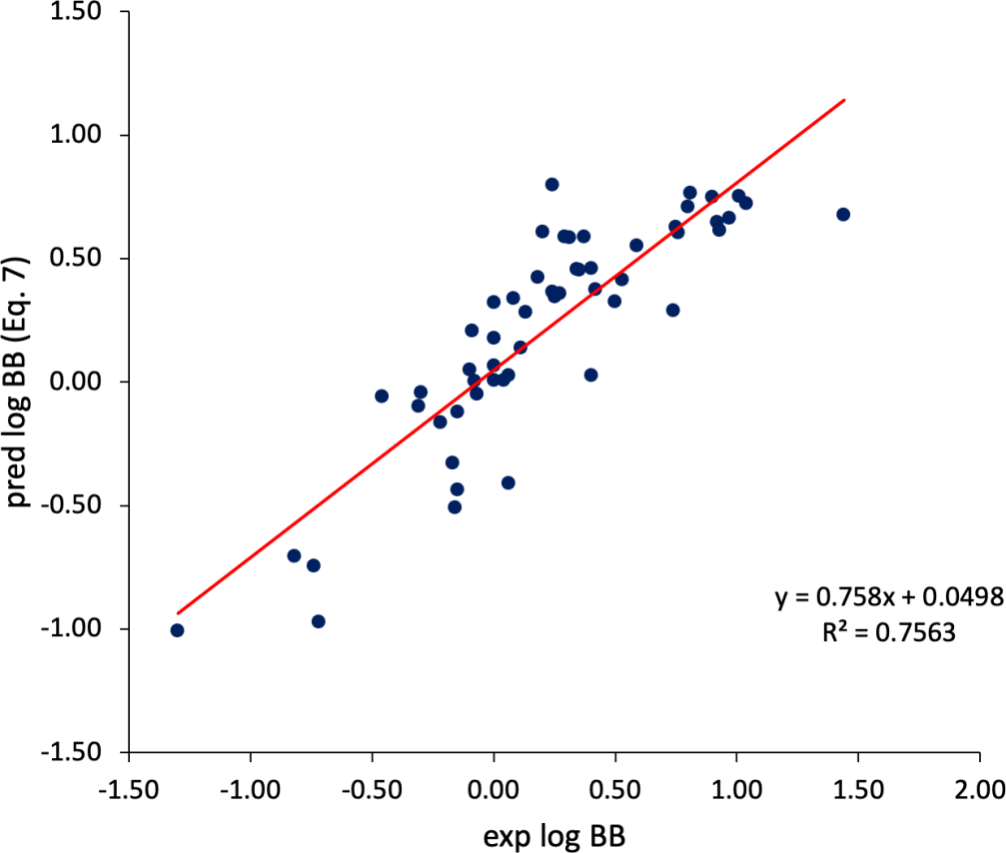
Experimental vs predicted log BB values calculated according to Eq. (7).

The statistics of the equations has been detailed in 2.3, while Exc identifies the compound that was excluded from the regression. According to both equations (6) and (7), the BBB diffusion of chemicals seems to be promoted by molecular lipophilicity and hindered for molecules featuring high cΔ/Δ’log *k*_w_^IAM.MG^, which is an index accounting for the excess of the polar/electrostatic interaction forces realized in the interplay between the chemical species and membrane phospholipids. Again, according to the models presented above, the BBB uptake of molecules is less efficient for those supporting many polar atoms (and specifically many hydroxyl groups). An *r*^2^ (n-1) value equal to 0.76 suggests that the statistic model is robust and reliable. A plot experimental *vs.* predicted (according to Eq. (7)) log BB values is presented in Figure 5. The value of each descriptor is reported in supporting information (Table S2).

## Conclusions

The present study proposes a method to streamline the drug discovery/development process and filter out solutes whose BBB permeation is envisaged to be substandard. Even if the dataset is limited in size and the method is not mature enough for broad implementation alone, it may be applied, along with other methodologies by pharmaceutical companies and research institutes, to focus only on candidates that tend to concentrate on the brain. This way, all the others can be neglected, thus saving time and money resources.



## Figures and Tables

**Table 1.** Names, chemical nature (A= acid, B=basic, BB= bibasic, N= neutral), calculated log *k*_w_^IAM.MG^ and log *k*_w_^IAM.DD2^, exp log *P*^N^, clog *P*^N^ values for the dataset considered.

molecule	nature	clog *k*_w_^IAM.MG^	clog *k*_w_^IAM.DD2^	exp log *P*^N^ [36]	clog *P*^N^(1) [26]	clog *P*^N^(2) [37]	clog *D*^7.4^ [37]
1,1,1-trichloroethane	N	1.063	1.247	2.49	2.45	2.04	
1,2-dimethylbenzene	N	1.400	1.635	3.12	3.16	2.98	
1,4-dimethylbenzene	N	1.421	1.599	3.15	3.15	2.98	
1,7-dimethylxanthine	A	-0.073	-0.001	-0.22	-0.63	0.09	0.09
1-chloro-2,2,2-trifluoroethane	N	0.811	0.774		1.82	1.86	
1-hydroxymidazolam	N	1.839	2.043		3.09	2.9	
2,2-dimethylbutane	N	1.283	1.578	3.82	3.74	2.85	
2-methylpentane	N	1.388	1.706	3.21	3.66	2.82	
3-methylhexane	N	1.561	1.949		4.18	3.21	
3-methylpentane	N	1.292	1.599	3.60	3.98	2.82	
4-hydroxymidazolam	N	1.950	2.191		3.05	3.35	
acetaminophen	N	0.184	0.302	0.91	0.51	1.09	
acetone	N	-0.247	-0.359	-0.24	-0.29	0.38	
aminopyrine	N	1.045	1.349	1.00	0.94	1.60	
amobarbital	A	0.899	1.181	2.07	1.87	1.86	1.60
antipyrine	N	0.901	1.139	0.56	1.18	1.61	
bretazenil	N	2.103	2.447		3.05	2.29	
cyclohexane	N	1.476	1.671	3.44		2.38	
cyclopropane	N	0.284	0.105	1.72	1.56	1.19	
Desmonomethylpromazine	B	2.287	2.703		4.28	3.68	
didanosine	A	-0.404	-0.294	-1.24	-1.26	-0.50	-1.06
diethylene glycol divinyl ether	N	-0.127	0.200	0.87	1.26	0.87	
enflurane	N	1.075	1.203	2.10	2.24	2.42	
ethanol	N	-0.534	-0.683	-0.31	-0.40	-0.22	
ethyl ether	N	0.162	0.308	0.89	1.12	0.70	
ethylbenzene	N	1.398	1.616	3.15	3.27	2.91	
flunitrazepam	N	1.621	1.739	2.06	2.20	2.58	
fluroxene	N	0.570	0.637		1.70	1.58	
halothane	N	1.165	1.300	2.30	2.50	1.97	
indinavir	BB	2.864	2.745	2.90	3.26	2.39	
isobutyl alcohol	N	0.045	0.169	0.76	0.60	0.65	
isoflurane	N	1.074	1.207		2.30	2.48	
isopropyl alcohol	N	-0.241	-0.243	0.05	0.04	0.19	
mesoridazine	B	2.640	3.027	3.90	3.83	3.41	
methoxyflurane	N	0.864	1.070	2.21	2.01	1.91	
methyl cyclopentane	N	1.140	1.347	3.37	3.15	2.31	
methyl ethyl ketone	N	0.047	0.057	0.29	0.41	1.01	
mirtazapine	B	1.969	2.287	2.90		3.38	
m-xylene	N	1.410	1.641	3.20	3.15	2.98	
nevirapine	N	1.152	1.332	2.50 [25]	1.75	2.19	
n-heptane	N	1.791	2.189			3.28	
n-hexane	N	1.545	1.861			2.88	
nordazepam	N	1.838	2.055		2.79	3.24	
northioridazine	B	3.120	3.607		5.29	5.1	
n-pentane	N	1.299	1.529			2.49	
quinidine	B	2.016	2.394	3.44	2.82	2.32	
sulforidazine	B	2.684	3.057	4.45	4.32	3.6	
teflurane	N	1.029	1.066		2.07	1.63	
thioridazine	B	3.318	3.816	5.90	5.93	5.48	
thioxolone	N	2.414	2.834	3.90	2.69	2.93	
tiotidine	B	0.186	0.375	0.68	0.59	1.18	
triazolam	N	2.102	2.365	2.42	2.94	3.31	
trichloroethylene	N	0.837	0.944		2.45	2.17	
trifluoperazine	BB	3.305	3.651	5.03	4.87	4.72	
valproic acid	A	1.135	1.542	2.75	2.54	2.61	0.37
zidovudine	A	-0.063	0.094	0.05	-0.1	-0.22	-0.28

**Table 2.** cΔ/Δ’log *k*_w_^IAM.MG^, cΔ/Δ’log *k*_w_^IAM.DD2^ values and experimental log BB values for the dataset considered.

molecule	cΔ/Δ’log *k*_w_^IAM.MG^	cΔ/Δ’log *k*_w_^IAM.DD2^	log BB
1,1,1-trichloroethane	-0.177	-0.196	0.40
1,2-dimethylbenzene	-0.339	-0.396	0.37
1,4-dimethylbenzene	-0.342	-0.460	0.31
1,7-dimethylxanthine	0.588	0.798	0.06
1-chloro-2,2,2-trifluoroethane	0.070	-0.080	0.08
1-hydroxymidazolam	0.274	0.217	-0.07
2,2-dimethylbutane	-1.010	-1.107	1.04
2-methylpentane	-0.422	-0.409	0.97
3-methylhexane	-0.249	-0.166	0.90
3-methylpentane	-0.827	-0.880	1.01
4-hydroxymidazolam	0.029	-0.055	-0.30
acetaminophen	0.195	0.335	-0.31
acetone	0.675	0.748	-0.15
aminopyrine	0.985	1.298	0.00
amobarbital	0.364	0.570	0.04
antipyrine	1.189	1.499	-0.10
bretazenil	1.021	1.191	-0.09
cyclohexane	-0.516	-0.659	0.92
cyclopropane	-0.346	-0.618	0.00
desmonomethylpromazine	0.104	0.149	0.59
didanosine	1.168	1.579	-1.30
diethylene glycol divinyl ether	-0.084	0.270	0.11
Enflurane	0.144	0.125	0.24
ethanol	0.444	0.490	-0.16
ethyl ether	0.189	0.360	0.00
ethylbenzene	-0.365	-0.443	0.20
flunitrazepam	0.721	0.698	0.06
fluroxene	0.051	0.044	0.13
halothane	0.075	0.035	0.35
indinavir	1.299	0.919	-0.74
isobutyl alcohol	0.175	0.342	-0.17
Isoflurane	0.174	0.166	0.42
isopropyl alcohol	0.451	0.593	-0.15
mesoridazine	0.283	0.267	-0.36
methoxyflurane	-0.154	-0.111	0.25
methyl cyclopentane	-0.797	-0.918	0.93
methyl ethyl ketone	0.549	0.669	-0.08
mirtazapine	0.404	0.461	0.53
m-xylene	-0.392	-0.465	0.29
nevirapine	-0.096	-0.120	0.00
n-heptane	-0.075	0.008	0.81
n-hexane	-0.004	0.054	0.80
nordazepam	0.004	-0.088	0.50
northioridazine	-0.187	-0.273	0.75
n-pentane	0.059	0.086	0.76
quinidine	0.024	0.064	-0.46
sulforidazine	-0.108	-0.216	0.18
teflurane	0.470	0.427	0.27
thioridazine	-0.623	-0.812	0.24
thioxolone	0.057	0.074	0.40
tiotidine	0.379	0.623	-0.82
triazolam	0.917	0.988	0.74
trichloroethylene	-0.150	-0.200	0.34
trifluoperazine	0.053	-0.164	1.44
valproic acid	1.574	2.079	-0.22
zidovudine	0.891	1.239	-0.72

**Table 3.** Variable considered and *r*^2^ *vs* log BB values. A detailed description of the descriptors is offered in supporting information (Table S1). The statistics of the each regressor is reported in 2.3.

Variable	*r* ^2^	*q^2^*	*SE*	*F*	*P*	*PC*
TPSA(NO)	0.46	0.42	0.385	46.08	9.08e-09	7.985
ALOGPS_logP	0.42	0.37	0.398	39.36	6.19e-08	8.561
nO	0.38	0.33	0.411	33.46	3.77e-07	9.138
*c*Δ*/*Δ*’log k_w_^IAM.DD2^*	0.38	0.32	0.413	32.82	5.36e-07	9.205
*c*Δ*/*Δ*’log k_w_^IAM.MG^*	0.37	0.32	0.414	32.36	5.36e07	9.255

## References

[1] Kennedy J.P. Williams L. Bridges T.M. Daniels R.N. Weaver D. Lindsley C.W.. Application of combinatorial chemistry science on modern drug discovery. J. Comb. Chem. 10 (2008) 345-354. https://dx.doi.org/10.1021/cc700187t. 10.1021/cc700187t 18220367

[2] Renaud J.P. Chung C.W. Danielson U.H. Egner U. Hennig M. Hubbard R.E. Nar H.. Biophysics in drug discovery: impact, challenges and opportunities. Nat. Rev. Drug Discov. 15 (2016) 679-698, https://dx.doi.org/10.1038/nrd.2016.123. 10.1038/nrd.2016.123 27516170

[3] Saeidnia S. Manayi A. Abdollahi M.. From in vitro Experiments to in vivo and Clinical Studies; Pros and Cons. Curr. Drug Discov. Technol. 12 (2015) 218-224. https://dx.doi.org/10.2174/1570163813666160114093140. 10.2174/1570163813666160114093140 26778084

[4] Zeiadeh I. Najjar A. Karaman R.. Strategies for Enhancing the Permeation of CNS-Active Drugs through the Blood-Brain Barrier: A Review. Molecules 23 (2018) 1289. https://dx.doi.org/10.3390/molecules23061289. 10.3390/molecules23061289 PMC6100436 29843371

[5] Patel M.M. Patel B.M.. Crossing the Blood-Brain Barrier: Recent Advances in Drug Delivery to the Brain. CNS Drugs 31 (2017) 109-133. https://dx.doi.org/10.1007/s40263-016-0405-9. 10.1007/s40263-016-0405-9 28101766

[6] Dong X.. Current Strategies for Brain Drug Delivery. Theranostics 8 (2018) 1481-1493. https://dx.doi.org/10.7150/thno.21254. 10.7150/thno.21254 29556336 PMC5858162

[7] Heymans M. Sevin E. Gosselet F. Lundquist S. Culot M.. Mimicking brain tissue binding in an in vitro model of the blood-brain barrier illustrates differences between in vitro and in vivo methods for assessing the rate of brain penetration. Eur. J. Pharm. Biopharm. 127 (2018) 453-461. https://dx.doi.org/10.1016/j.ejpb.2018.03.007. 10.1016/j.ejpb.2018.03.007 29602020

[8] Chrysanthakopoulos M. Tsopelas F. Tsantili-Kakoulidou A.. Biomimetic Chromatography: A Useful Tool in the Drug Discovery Process. Adv. Chromatogr. 51 (2014) 91-125. 26462371

[9] Russo G. Ermondi G. Caron G. Verzele D. Lynen F.. Into the first biomimetic sphingomyelin stationary phase: Suitability in drugs’ biopharmaceutic profiling and block relevance analysis of selectivity. Eur. J. Pharm. Sci. 156 (2021) 105585. https://dx.doi.org/10.1016/j.ejps.2020.105585. 10.1016/j.ejps.2020.105585 33045369

[10] Stergiopoulos C. Tsopelas F. Valko K.. Prediction of hERG inhibition of drug discovery compounds using biomimetic HPLC measurements. ADMET and DMPK 9 (2021) 191-207. https://doi.org/10.5599/admet.995. 10.5599/admet.995 PMC8920097 35300361

[11] Valko K.L. Zhang T.. Biomimetic properties and estimated in vivo distribution of chloroquine and hydroxy-chloroquine enantiomers. ADMET and DMPK 9 (2021) 151-165. https://doi.org/10.5599/admet.929. 10.5599/admet.929 PMC8920107 35299770

[12] Tsopelas F. Vallianatou T. Tsantili-Kakoulidou A.. Advances in immobilized artificial membrane (IAM) chromatography for novel drug discovery. Expert Opin. Drug Discov. 11 (2016) 473-488. https://dx.doi.org/10.1517/17460441.2016.1160886. 10.1517/17460441.2016.1160886 26966996

[13] Russo G. Grumetto L. Szucs R. Barbato F. Lynen F.. Screening therapeutics according to their uptake across the blood-brain barrier: A high throughput method based on immobilized artificial membrane liquid chromatography-diode-array-detection coupled to electrospray-time-of-flight mass spectrometry. Eur. J. Pharm. Biopharm. 127 (2018) 72-84. https://dx.doi.org/10.1016/j.ejpb.2018.02.004. 10.1016/j.ejpb.2018.02.004 29427629

[14] Grumetto L. Carpentiero C. Barbato F.. Lipophilic and electrostatic forces encoded in IAM-HPLC indexes of basic drugs: their role in membrane partition and their relationships with BBB passage data. Eur. J. Pharm. Sci. 45 (2012) 685-692. https://dx.doi.org/10.1016/j.ejps.2012.01.008. 10.1016/j.ejps.2012.01.008 22306648

[15] Grumetto L. Carpentiero C. Di Vaio P. Frecentese F. Barbato F.. Lipophilic and polar interaction forces between acidic drugs and membrane phospholipids encoded in IAM-HPLC indexes: their role in membrane partition and relationships with BBB permeation data. J. Pharm. Biomed. Anal. 75 (2013) 165-172, https://dx.doi.org/10.1016/j.jpba.2012.11.034. 10.1016/j.jpba.2012.11.034 23261809

[16] Grumetto L. Russo G. Barbato F.. Indexes of polar interactions between ionizable drugs and membrane phospholipids measured by IAM-HPLC: their relationships with data of Blood-Brain Barrier passage. Eur J Pharm Sci 65 (2014) 139-146. https://dx.doi.org/10.1016/j.ejps.2014.09.015. 10.1016/j.ejps.2014.09.015 25262853

[17] Grumetto L. Russo G. Barbato F.. Relationships between human intestinal absorption and polar interactions drug/phospholipids estimated by IAM-HPLC. Int. J. Pharm. 489 (2015) 186-194. https://dx.doi.org/10.1016/j.ijpharm.2015.04.062. 10.1016/j.ijpharm.2015.04.062 25917756

[18] Grumetto L. Russo G. Barbato F.. Immobilized Artificial Membrane HPLC Derived Parameters vs PAMPA-BBB Data in Estimating in Situ Measured Blood-Brain Barrier Permeation of Drugs. Mol. Pharm. 13 (2016) 2808-2816. https://dx.doi.org/10.1021/acs.molpharmaceut.6b00397. 10.1021/acs.molpharmaceut.6b00397 27377191

[19] Grumetto L. Russo G. Barbato F.. Polar interactions drug/phospholipids estimated by IAM-HPLC vs cultured cell line passage data: Their relationships and comparison of their effectiveness in predicting drug human intestinal absorption. Int. J. Pharm. 500 (2016) 275-290. https://dx.doi.org/10.1016/j.ijpharm.2016.01.019. 10.1016/j.ijpharm.2016.01.019 26780120

[20] Braumann T. Weber G.. Grimme L.. Quantitative structure—activity relationships for herbicides: Reversed-phase liquid chromatographic retention parameter, log kw, versus liquid-liquid partition coefficient as a model of the hydrophobicity of phenylureas, s-triazines and phenoxycarbonic acid derivatives. Journal of Chromatography A 261 (1983) 329-343. https://doi.org/10.1016/S0021-9673(01)87961-9. 10.1016/S0021-9673(01)87961-9

[21] Carpenter T.S. Kirshner D.A. Lau E.Y. Wong S.E. Nilmeier J.P. Lightstone F.C.. A method to predict blood-brain barrier permeability of drug-like compounds using molecular dynamics simulations. Biophys. J. 107 (2014) 630-641. https://dx.doi.org/10.1016/j.bpj.2014.06.024. 10.1016/j.bpj.2014.06.024 25099802 PMC4129472

[22] Russo G. Barbato F. Grumetto L. Philippe L. Lynen F. Goetz G.H.. Entry of therapeutics into the brain: Influence of exposed polarity calculated in silico and measured in vitro by supercritical fluid chromatography. Int. J. Pharm. 560 (2019) 294-305. https://dx.doi.org/10.1016/j.ijpharm.2019.02.008. 10.1016/j.ijpharm.2019.02.008 30771469

[23] Pandey P.K. Sharma A.K. Gupta U.. Blood brain barrier: An overview on strategies in drug delivery, realistic in vitro modeling and in vivo live tracking. Tissue Barriers 4 (2016) e1129476. https://dx.doi.org/10.1080/21688370.2015.1129476. 10.1080/21688370.2015.1129476 27141418 PMC4836458

[24] Russo G. Grumetto L. Barbato F. Vistoli G. Pedretti A.. Prediction and mechanism elucidation of analyte retention on phospholipid stationary phases (IAM-HPLC) by in silico calculated physico-chemical descriptors. Eur. J. Pharm. Sci. 99 (2017) 173-184. https://dx.doi.org/10.1016/j.ejps.2016.11.026. 10.1016/j.ejps.2016.11.026 27919703

[25] Wishart D.S. Feunang Y.D. Guo A.C. Lo E.J. Marcu A. Grant J.R. Sajed T. Johnson D. Li C. Sayeeda Z. Assempour N. Iynkkaran I. Liu Y. Maciejewski A. Gale N. Wilson A. Chin L. Cummings R. Le D. Pon A. Knox C. Wilson M.. DrugBank 5.0: a major update to the DrugBank database for 2018. Nucleic Acids Res. 46 (2018) D1074-D1082. https://dx.doi.org/10.1093/nar/gkx1037. 10.1093/nar/gkx1037 29126136 PMC5753335

[26] Tetko I.V. Tanchuk V.Y.. Application of associative neural networks for prediction of lipophilicity in ALOGPS 2.1 program. J. Chem. Inf. Comput. Sci. 42 (2002) 1136-1145. https://dx.doi.org/10.1021/ci025515j. 10.1021/ci025515j 12377001

[27] MarvinSketch version 17.1.16.0, ChemAxon Ltd, Budapest, Hungary http://www.chemaxon.com (2017).

[28] Platts J.A. Abraham M.H. Zhao Y.H. Hersey A. Ijaz L. Butina D.. Correlation and prediction of a large blood-brain distribution data set--an LFER study. Eur. J. Med. Chem. 36 (2001) 719-730. https://dx.doi.org/10.1016/s0223-5234(01)01269-7. 10.1016/s0223-5234(01)01269-7 11672881

[29] Griffin W.C.. Calculation of HLB Values of Non-ionic Surfactants. J. Soc. Cosmet. Chem. 5 (1954) 249-256.

[30] Davies J.. A QUANTITATIVE KINETIC THEORY OF EMULSION TYPE . I . PHYSICAL CHEMISTRY OF THE EMULSIFYING. 2003.

[31] Molinspiration: log P n-octanol-water partition coefficient. http://www.molinspiration.com/services/logp.html. 2021.

[32] Mauri A. Consonni V. Todeschini R., Molecular Descriptors, in: Leszczynski J. Kaczmarek-Kedziera A. Puzyn T. Papadopoulos M. G. Reis H. Shukla M. K. (Eds.) Handbook of Computational Chemistry, Springer International Publishing, Cham, 2017, pp. 2065-2093. https://dx.doi.org/10.1007/978-3-319-27282-5_51. 10.1007/978-3-319-27282-5_51

[33] Pedretti A. Villa L. Vistoli G.. VEGA-an open platform to develop chemo-bio-informatics applications, using plug-in architecture and script programming. J. Comput. Aided Mol. Des. 18 (2004) 167-173. https://dx.doi.org/ 10.1023/b:jcam.0000035186.90683.f2. 10.1023/b:jcam.0000035186.90683.f2 15368917

[34] Russo G. Grumetto L. Szucs R. Barbato F. Lynen F.. Determination of in Vitro and in Silico Indexes for the Modeling of Blood-Brain Barrier Partitioning of Drugs via Micellar and Immobilized Artificial Membrane Liquid Chromatography. J. Med. Chem. 60 (2017) 3739-3754. https://dx.doi.org/10.1021/acs.jmedchem.6b01811. 10.1021/acs.jmedchem.6b01811 28399367

[35] Verma J. Khedkar V.M. Coutinho E.C.. 3D-QSAR in drug design--a review. Curr. Top. Med. Chem. 10 (2010) 95-115. https://dx.doi.org/10.2174/156802610790232260. 10.2174/156802610790232260 19929826

[36] Kim S. Chen J. Cheng T. Gindulyte A. He J. He S. Li Q. Shoemaker B.A. Thiessen P.A. Yu B. Zaslavsky L. Zhang J. Bolton E.E.. PubChem in 2021: new data content and improved web interfaces. Nucleic Acids Research 49 (2020) D1388-D1395. https://dx.doi.org/10.1093/nar/gkaa971. 10.1093/nar/gkaa971 PMC7778930 33151290

[37] M.S.f.W.-b.P.v. http://www.chemaxon.com/products/marvin/marvinsketch/. Calculation module designed by Chemaxon.

[38] Tsopelas F. Vallianatou T. Tsantili-Kakoulidou A.. Advances in immobilized artificial membrane (IAM) chromatography for novel drug discovery. Expert Opinion on Drug Discovery 11 (2016) 473-488. https://dx.doi.org/10.1517/17460441.2016.1160886. 10.1517/17460441.2016.1160886 26966996

[39] Taillardat-Bertschinger A. Barbato F. Quercia M.T. Carrupt P.-A. Reist M. La Rotonda M.I. Testa B.. Structural Properties Governing Retention Mechanisms on Immobilized Artificial Membrane (IAM) HPLC Columns. Helvetica Chimica Acta 85 (2002) 519-532. https://dx.doi.org/10.1002/1522-2675(200202)85:2<519::Aid-hlca519>3.0.Co;2-q. 10.1002/1522-2675(200202)85:2<519::Aid-hlca519>3.0.Co;2-q

[40] Arnott J.A. Planey S.L.. The influence of lipophilicity in drug discovery and design. Expert Opin. Drug Discov. 7 (2012) 863-875. https://dx.doi.org/10.1517/17460441.2012.714363. 10.1517/17460441.2012.714363 22992175

[41] Wan H. Holmen A.G.. High throughput screening of physicochemical properties and in vitro ADME profiling in drug discovery. Comb. Chem. High Throughput Screen. 12 (2009) 315-329. https://dx.doi.org/10.2174/138620709787581701. 10.2174/138620709787581701 19275537

[42] Chen Z. Weber S.G.. High-throughput method for lipophilicity measurement. Anal. Chem. 79 (2007) 1043-1049. https://dx.doi.org/10.1021/ac061649a. 10.1021/ac061649a 17263333 PMC2538958

[43] Jeffrey P. Summerfield S.G.. Challenges for blood-brain barrier (BBB) screening. Xenobiotica 37 (2007) 1135-1151. https://dx.doi.org/10.1080/00498250701570285. 10.1080/00498250701570285 17968740

[44] Diamond J.M. Wright E.M. Smyth D.H.. Molecular forces governing non-electrolyte permeation through cell membranes. Proceedings of the Royal Society of London. Series B. Biological Sciences 172 (1969) 273-316. https://dx.doi.org/10.1098/rspb.1969.0022. 10.1098/rspb.1969.0022 4388931

[45] Chikhale E.G. Ng K.-Y. Burton P.S. Borchardt R.T.. Hydrogen Bonding Potential as a Determinant of the in Vitro and in Situ Blood–Brain Barrier Permeability of Peptides. Pharmaceutical Research 11 (1994) 412-419. https://dx.doi.org/10.1023/A:1018969222130. 10.1023/A:1018969222130 8008709

