# Supplementary material for: cΔlog kwIAM: can we afford estimation of small molecules’ blood-brain barrier passage based upon in silico phospholipophilicity?
Source: ADMET DMPK. 2021 Dec 15;9(4):267–81. doi: 10.5599/admet.1034 (PMC8920103; doi:10.5599/admet.1034)
Supplement: Supplementary file 1 [file admet-9-1034-S1.pdf]

Supporting information

cΔlog k_w^{IAM}: can we afford estimation of small molecules' blood-brain barrier passage based upon *in silico* phospholipophilicity?

*Lucia Grumetto[1], Giacomo Russo[2]**

1. Pharm-Analysis & Bio-Pharm Laboratory, Department of Pharmacy, School of Medicine and Surgery, University of Naples Federico II, Via D. Montesano, 49, I-80131, Naples, Italy.

2. School of Applied Sciences, Sighthill Campus, Edinburgh Napier University, 9 Sighthill Ct, EH11 4BN Edinburgh, United Kingdom.

*Corresponding Author: E-mail: G.Russo@napier.ac.uk; Tel.: +44 (0) 131 455 3464; Fax: +44 (0) 131 455 3555

Table S1. Definition of the physico-chemical descriptors reported in the manuscript

Descriptor	Definition
miLogP	octanol-water partition coefficient (log P) calculated by Molinspiration Cheminformatics (1)
Heavy Atoms	Number of heavy atoms
HLB_M	Mean of HLB_{PSA} values, HLB values calculated according to Griffin's method(2) and HLB values calculated according to Davies's method(3)
HLB_{PSA}	hydrophilic-lipophilic balance (HLB) calculated according to the following: $HLB_{PSA} = 20 * \frac{PSA}{Surface}$ in which PSA is the polar surface area and Surface is the total surface
Rotatable bonds	Number of rotatable bonds
TPSA(NO)	topological polar surface area using N,O polar contributions
ALOGPS_logP	octanol-water partition coefficient (log P) calculated by the software ALOGPS 2.1(4)
nO	Number of oxygen atoms
TPSA(Tot)	topological polar surface area using N,O,S,P polar contributions
nROH	number of hydroxyl groups

Table S2. Descriptors employed in the multiple linear regression.

Molecule	nROH	TPSA(Tot)	ALOGPS_logP
**1,1,1-trichloroethane**	0	0	2.45
**1,2-dimethylbenzene**	0	0	3.16
**1,4-dimethylbenzene**	0	0	3.15
**1,7-dimethylxanthine**	0	72.68	-0.63
**1-chloro-2,2,2-trifluoroethane**	0	0	1.82
**1-Hydroxymidazolam**	1	50.41	3.09
**2,2-dimethylbutane**	0	0	3.74
**2-methylpentane**	0	0	3.6
**3-methylhexane**	0	0	4.18
**3-methylpentane**	0	0	3.98
**4-Hydroxymidazolam**	1	50.41	3.05
**acetaminophen**	0	49.33	0.51
**acetone**	0	17.07	-0.29
**aminopyrine**	0	30.17	0.94
**amobarbital**	0	75.27	1.87
**antipyrine**	0	26.93	1.18
**Bretazenil**	0	64.43	3.05
**cyclohexane**	0	0	3.46
**cyclopropane**	0	0	1.56
**desmonomethylpromazine**	0	45.2	4.28
**didanosine**	1	93.03	-1.26
**Diethylene glycol divinyl ether**	0	27.69	1.26
**enflurane**	0	9.23	2.24
**ethanol**	1	20.23	-0.4
**ethyl ether**	0	9.23	1.12
**ethylbenzene**	0	0	3.27
**flunitrazepam**	0	78.49	2.2
**Fluroxene**	0	9.23	1.7
**halothane**	0	0	2.5
**indinavir**	2	118.03	3.26
**isobutyl alcohol**	1	20.23	0.6
**isoflurane**	0	9.23	2.3
**Isopropyl alcohol**	1	20.23	0.04
**mesoridazine**	0	72.69	3.83
**methoxyflurane**	0	9.23	2.01
**methyl cyclopentane**	0	0	3.15
**methyl ethyl ketone**	0	17.07	0.41
**mirtazapine**	0	19.37	2.9
**m-xylene**	0	0	3.15
**nevirapine**	0	63.57	1.75
**N-heptane**	0	0	4.33
**N-hexane**	0	0	4.02
**nordazepam**	0	41.46	2.79

**Northioridazine**	0	70.5	5.29
**N-pentane**	0	0	3.41
**Quinidine**	1	45.59	2.82
**Sulforidazine**	0	78.93	4.32
**Teflurane**	0	0	2.07
**thioridazine**	0	61.71	5.93
**thioxolone**	0	78.68	1.97
**Tiotidine**	0	191.04	0.59
**triazolam**	0	43.07	2.94
**trichloroethylene**	0	0	2.45
**trifluoperazine**	0	39.65	4.87
**Valproic acid**	1	37.3	2.54
**zidovudine**	1	133.08	-0.1

REFERENCES

1. Molinspiration: log P n-octanol-water partition coefficient. http://www.molinspiration.com/services/logp.html. 2021 [Available from: http://www.molinspiration.com/services/logp.html.
2. Griffin WC. Calculation of HLB Values of Non-ionic Surfactants. J Soc Cosmet Chem. 1954;5:249-56.
3. Davies J, editor A QUANTITATIVE KINETIC THEORY OF EMULSION TYPE . I . PHYSICAL CHEMISTRY OF THE EMULSIFYING2003.
4. Tetko IV, Tanchuk VY. Application of Associative Neural Networks for Prediction of Lipophilicity in ALOGPS 2.1 Program. Journal of Chemical Information and Computer Sciences. 2002;42(5):1136-45.
